# Probing functional polymorphisms in the dengue vector, *Aedes aegypti*

**DOI:** 10.1186/1471-2164-14-739

**Published:** 2013-10-29

**Authors:** Mariangela Bonizzoni, Monica Britton, Osvaldo Marinotti, William Augustine Dunn, Joseph Fass, Anthony A James

**Affiliations:** 1 Program in Public Health, University of California, Irvine, CA 92697, USA; 2 Department of Molecular Biology and Biochemistry, University of California, Irvine, CA 92697, USA; 3 Bioinformatics Core of the UC Davis Genome Center, University of California, Davis, CA 95616, USA; 4 Department of Microbiology and Molecular Genetics, University of California, Irvine, CA 92697, USA

**Keywords:** Mosquito, Variation, RNA-seq, SNP, Immunity

## Abstract

**Background:**

Dengue is the most prevalent arboviral disease world-wide and its primary vector is the mosquito *Aedes aegypti*. The current lack of commercially-available vaccines makes control of vector populations the only effective strategy to prevent dengue transmission. *Aedes aegypti* geographic populations exhibit great variability in insecticide resistance and susceptibility to dengue infection. The characterization of single nucleotide polymorphisms (SNPs) as molecular markers to study quantitatively this variation is needed greatly because this species has a low abundance of microsatellite markers and limited known restriction fragments length polymorphisms (RFLPs) and single-strand conformation polymorphism (SSCP) markers.

**Results:**

We used RNA-seq to characterize SNPs in three *Ae. aegypti* strains, including the Liverpool (LVP) strain, from which the current genome annotation is derived. We identified 131,764 unique genome locations with at least one alternative nucleotide to what is reported in the reference annotation. These comprised changes in both open-reading frames (ORFs) and untranslated regions (UTRs) of transcripts*.* An in depth-look at sequence variation in immunity genes revealed that those associated with autophagy, MD2-like receptors and Peptidoglycan Recognition Proteins had more sequence variation in their 3’UTRs than mutations associated with non-synonymous changes. This supports the conclusion that these genes had maintained their functional specificity while being adapted to different regulatory domains. In contrast, a number of peroxidases, serpins and Clip-domain serine proteases exhibited conservation of putative UTR regulatory sequences while displaying diversification of the ORFs. Transcriptome evidence also was found for ~2500 novel transcriptional units (NTUs) not annotated in the reference genome.

**Conclusions:**

The transcriptome-wide assessment of within and inter-strain polymorphisms in *Ae. aegypti* adds considerably to the number of molecular markers available for genetic studies in this mosquito. Additionally, data supporting NTU discovery emphasizes the need for continuous amendments of the reference genome annotation.

## Background

Approximately 40% of the global human population is threatened by dengue epidemics, making it the most prevalent arboviral disease world-wide [1]. The main vector of dengue is the cosmopolitan mosquito, *Aedes aegypti*. Control of vector populations remains the primary line of defense for disease prevention due to the lack of a vaccine and effective antiviral drugs. Successful deployment of vector control strategies with classical tools (i.e. insecticides) [2] and novel control strategies based on genetically-modified mosquitoes requires knowledge of the genetic structure of mosquito populations.

Considerable genetic variation in different traits has been documented in geographically-distinct *Ae. aegypti* populations, including variability in genes that determine insecticide resistance and vector competence [3]. The study of genetic variation requires molecular markers [4]. *Aedes aegypti* has a low abundance of microsatellite markers and limited (less than 200) known restriction fragments length polymorphisms (RFLPs) and single-strand conformation polymorphism (SSCP) markers [5-7]. Consequently, the characterization of molecular markers is needed greatly in *Ae. aegypti*.

RNA-seq is a reliable methodology to identify single nucleotide polymorphisms (SNPs) and has been used to do so in a number of species [8-12]. The standard RNA-seq library preparation protocols target poly-adenylated RNAs, thus restricting detection of SNPs to sequences encoding open-reading frames (ORFs) and transcript untranslated regions (UTRs). As a consequence, RNA-seq approaches focus on “functional polymorphisms” and are more likely to identify adaptive rather than neutral genetic variability [13]. Changes in open reading frames can affect protein sequences and consequently their structures and functions, while polymorphisms in UTRs can alter regulatory elements or miRNA binding sites influencing mRNA stability and/or translation [14,15].

We used RNA-seq to identify sequence variation in the transcriptomes of three *Ae. aegypti* strains, Liverpool (LVP), Chetumal (CTM) and Rexville D-Puerto Rico (RexD). LVP is a long-standing laboratory-adapted strain and was used to generate the *Ae. aegypti* reference-genome [16]. The CTM strain was derived in the early 2000s from mosquitoes collected in Chetumal, Mexico [17]. RexD originated in the early 1990s from mosquitoes collected in Puerto Rico [18]. Our results provide insights into functional sequence variability in *Ae. aegypti.*

## Results

### RNA-seq data summary

**Figure 1:**
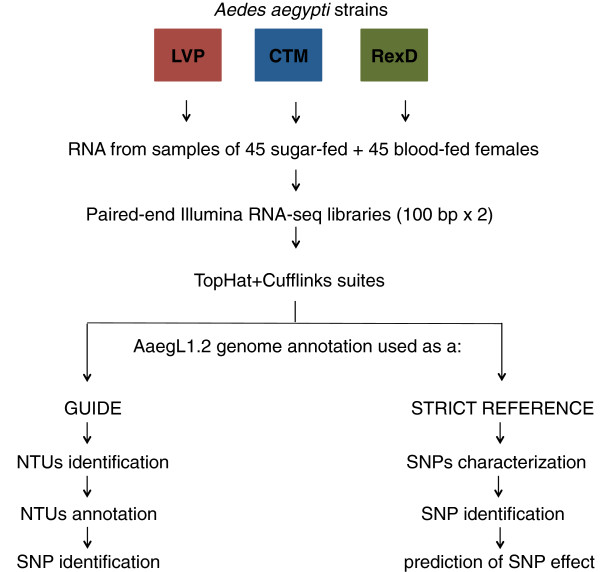
**Schematic representation of the design and analysis pipeline.** Three *Ae. aegypti* strains, LVP, CTM and RexD, were used to generate one RNA-seq library each starting from total RNA of 90 mosquitoes (45 blood-fed and 45 sugar-fed). Approximately 100 nucleotides were sequenced at both ends of each cDNA using Illumina technology. RNA-seq libraries were analyzed using the TopHat/Cufflinks suite with the AaegL1.2 genome annotation. The AaegL1.2 genome annotation was used as guide for gene annotation in Cufflinks, which was run to allow the identification of novel transcriptional units (NTUs). NTUs were annotated by Blast2GO and SNPs identified by Freebayes. SNPs in annotated genes were identified by Freebayes. The effect of the SNPs on the predicted coding sequence was identified by snpEff using the AaegL1.2 genome annotation as a strict reference for gene annotation.

Paired-end Illumina RNA-seq libraries were generated from RNA samples extracted from LVP, CTM or RexD mosquitoes. RNA samples from 90 mosquitoes (180 haploid genomes), represented equally by sugar- and blood-fed females, were used for each library (Figure 1). Paired-end sequencing of each cDNA library generated total trimmed sequences of 22.5, 20.7 and 38.3 gigabase pairs (Gb) in length for CTM, RexD and LVP, respectively, with specific gene representation dependent on its expression level in the samples used to prepare the RNA.

### SNP identification and classification

#### Characterization of genes for in-depth SNP analyses

A total of 11,301 genes were found with RNA-seq read coverage ≥ 90% of their transcript length in all three strains using Cufflinks and the *Ae. aegypti* genome annotation (AaegL1.2) as a strict reference. These genes had 119,608 polymorphic sites (PS) in LVP; 213,604 in CTM and 221,951 in RexD. Polymorphic sites are loci with a nucleotide other than that assigned in the consensus reference genome [16]. Polymorphic sites may include more than one allele because the RNA-seq data were generated from pooled RNA samples derived from 90 mosquitoes/strain. Furthermore, some sites may be incorporated in more than one gene depending on gene orientation (5’- or 3’-end overlap) and location (proximity).

We used read-coverage as a measure for nucleotide variant-calling confidence. Depth of coverage was expressed in Fragments Per Kilobase of exon model per Million mapped fragments (FPKM). An arbitrary cut-off of 15 FPKM, which is considered a moderate abundance level [19,20], was used to identify genes for analyses of sequence polymorphisms. A total of 5113, 5059 and 5154 genes had FPKM > 15 in LVP, CTM and RexD, respectively, with 4492 common to all three strains (Additional file 1: Figure S1). The 4492 genes that in all three strains have RNA-seq coverage over ≥ 90% of their transcript length and FPKM > 15 are referred to hereafter as “SNP-genes (genes showing polymorphic sites)” (Additional file 2: Table S1).

#### Properties of SNP-genes and polymorphic sites

SNP-genes are distributed over ~57% (928/1636) of the *Ae. aegypti* supercontigs that have annotated genes (Additional file 3: Table S2). The majority (~55% [392/708]) of supercontigs with no SNP-genes has only one annotated gene, but 17 had ≥ 10 annotated genes. Specifically, supercontig 1.361 has 50 annotated genes, all of which are described as encoding structural constituents of cuticle. Supercontig 1.429 has 25 annotated genes, 12 of which encode cuticle constituents, eight are hypothetical proteins and the remaining five are associated with diverse functions such as metabolism, transcription, protein kinases, extracellular matrix glycoproteins and low-density lipoprotein receptors. Fifteen of the supercontigs with no SNP-genes (1.455, 1.847, 1.930, 1.1003, 1.1095, 1.26, 1.491, 1.681, 1.1101, 1.536, 1.846, 1.606, 1.676, 1.738 and 1.743) have 10–14 annotated genes that are predicted to encode hypothetical proteins or are associated with diverse functions including lipid metabolism, transport, nucleic acid binding, structural constituents of cuticle and oxido-reductase. These results indicate that genes encoding cuticular proteins are under-represented in the SNP-genes. Cuticular proteins are synthesized primarily by epidermal cells and their respective genes are transcribed during the larval stages [21]. This expression pattern may account for their absence or limited detection (0 ≤ FPKM < 15) when preparing RNA-seq libraries from samples of RNA derived from adults. Similarly, other genes with tissue-specific or developmentally-regulated expression may not be included among the SNP-genes. However, the numbers of annotated genes and SNP-genes per supercontig have a positive correlation (R2 = 0.795) (Additional file 3: Table S2), supporting the conclusion that these results and analyses can be generalized to the whole *Ae. aegypti* genome and transcriptome.

The 4492 SNP-genes had a total of 131,764 unique polymorphic sites with an alternative allele in at least one strain. Totals of 50,674, 91,326 and 94,323 polymorphic sites were identified in SNP-genes in LVP, CTM and RexD, respectively. This gives a density of polymorphic sites per 1000 bp (PS/kb) of 5.05, 9.13 and 9.43 in LVP, CTM and RexD, respectively. The density of polymorphic sites was not dependent on FPKM, indicating that SNP detection was not biased by RNA-seq coverage (Additional file 4: Figure S2). Pairwise comparisons of PS/kb between corresponding supercontigs of two strains showed a significant difference in density distribution between LVP and both CTM and RexD, but not between CTM and RexD (P value < 0.01).

A total of 28 genes meeting SNP-gene criteria had no polymorphisms, supporting the conclusion that these genes encode proteins with vital cellular functions (Additional file 5: Table S3). Remarkably, half of these encode proteins with unknown functions while the others are associated with metabolic functions, transport or transcription/translation. One (AAEL017211) of these 28 genes encodes the antimicrobial peptide cecropin. Expression levels for all of these non-polymorphic genes were higher than 20 FPKM, with eight having FPKM > 100 in all strains, revealing that undetected polymorphisms, if existent, occur at a low frequency in the studied *Ae. aegypti* strains.

### Effect of SNPs

The program SnpEff 3.0 was used to analyse the SNPs within the 131,764 polymorphic sites identified and assess their effects on ORFs and UTRs. Results are summarised by gene; consequently a SNP may be counted more than once if it is included in the ORFs or UTRs of overlapping genes (Additional file 6: Table S4). Most of the changes were associated with synonymous mutations, followed by changes in 3’UTRs. Between 15 and 29% of all detected SNPs were strain-specific (Table 1).

**Table 1:** **Predicted SNP effects on open-reading frames (ORFs) and untranslated regions (UTRs) of annotated SNP-genes**^**1**^

	**LVP**		**CTM**		**RexD**	
**Polymorphism class**	**SNPs**^**2**^	**Genes**	**SNPs**^**2**^	**Genes**	**SNPs**^**2**^	**Genes**
Non-synonymous	6577 (1108)	2296	11,815 (3553)	3072	12327 (3757)	3124
Splice site acceptor	180 (91)	168	160 (56)	146	137 (39)	126
Splice site donor	168 (89)	152	128 (46)	115	119 (32)	105
Start gained^3^	110 (13)	101	214 (70)	194	197 (52)	180
Start lost	10 (3)	10	20 (6)	19	17 (3)	17
Stop gained	34 (12)	33	52 (20)	49	58 (51)	42
Stop lost	26 (3)	23	38 (9)	35	31 (7)	29
Synonymous	29802 (3902)	3312	55691 (15287)	4111	57484 (15887)	4128
Synonymous stop	23 (2)	23	36 (11)	36	42 (14)	42
3'UTR	11,354 (2100)	2625	18,349 (5111)	3003	19,065 (5283)	3054
5'UTR	2844 (486)	1223	5133 (1506)	1709	5169 (1461)	1741

^1^Total number of SNPs predicted to induce non-synonymous and synonymous changes, splice site acceptors and donors, gain or loss of an initiation or termination codon within the open reading frame and changes in 3’- and 5’-UTRs are reported for each strain along with the number of genes in which they occur.

^2^The number of SNPs unique for each strain is in parenthesis.

^3^Only ATG start codons are included.

#### Sequence variation in immunity-related SNP-genes

**Figure 2:**
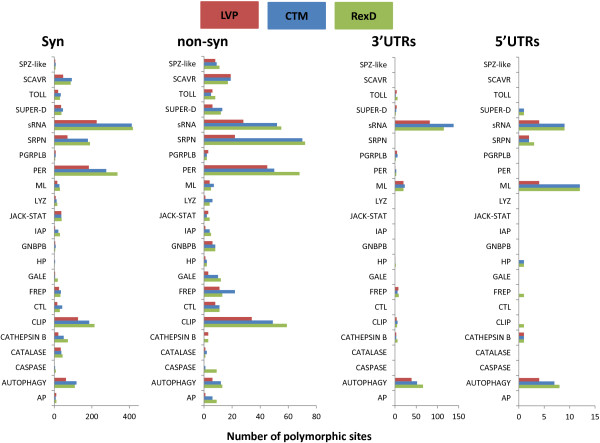
**Effect of SNPs on immunity genes.** Number of mutations associated with synonymous (syn), non-synonymous (non-syn) changes or located in 3’ or 5’UTRs (3’UTRS and 5’UTRs, respectively) detected in each strain across the immunity genes classified into the same functional class. Abbreviations: AP (Antimicrobial-peptide), AUTOPHAGY (autophagy-related genes); CASPASE; CATALASE, CATHEPSIN B, CLIP (Clip-domain serine protease); CTL (C-type lectin); FREP (fibrinogen-related protein); GALE (galectins); HP (putative function associated with immunity); GNBPBP (1,3-BETA-d Glucan Binding Proteins or Gram-negative binding protein); IAP (inhibitor of apoptosis); JACK-STAT (Jack-Stat pathway members); LYZ (Lysozyme); ML (MD2-like proteins); PER (peroxidase), PGRPLB (peptidoglycan recognition protein); SCAVR (Scavenger Receptors); SRPN (serpin); sRNA (small regulatory RNA pathway members), SUPER-D (super-oxido dismutase); TOLL (Toll pathway) and SPZ-like (*speatzle*-like).

A total of 124 immunity-related genes are among the SNP-genes. SNPs associated with non-synonymous mutations were prevalent in genes encoding B family CLIPs, SUPER-D, serpins, FREPs (AAEL006704 [FREP33]; AAEL006704 [FREP18] and AAEL007942 [FREP14]), C-type lectins, PER of the HPX family, caspase (AAEL005956 [CASPS16], Gram-negative binding proteins (GNBP) (AAEL007064 [GNBPB6], and GALEs (AAEL003541 [GALE1], AAEL003844 [GALE5], AAEL009842 [GALE12]) (Figure 2, Additional file 7: Table S5).

Several functional categories of immunity-related SNP-genes showed frequent polymorphisms in 3’UTRs, and these are often strain-specific. These categories include: 1) autophagy-related genes such as APG3 (AAEL000955), APG8 (AAEL007162), APG4B (AAEL007228), APG9 (AAEL009105), and DEBCL (AAEL001515), 2) ML-encoding genes such as ML2 (AAEL012064), ML13 (AAEL006854) ML21 (AAEL009760), and ML1 (AAEL004120) and 3) the genes RM62H (AAEL010787) and RM62B (AAEL001769), members of the PIWI pathway and LOQS (AAEL008687), a member of the miRNA pathway (Additional file 7: Table S5).

### Evidence for novel transcriptional units

**Figure 3:**
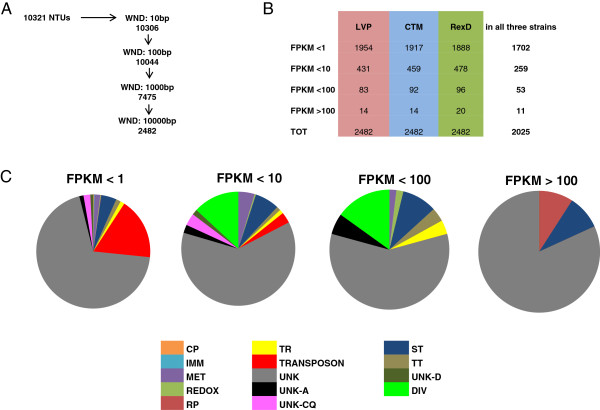
**Novel Transcriptional Units. A)** total number of novel transcriptional units (NTUs) identified using the *Ae. aegypti* genome annotation *(*AaegL1.2*)* (left column) as a guide in mapping RNA-seq reads and number of NTUs not within specific distance (WND) in base-pairs (bp) of known exons (right column). **B)** the 2482 NTUs identified outside a 10,000 bp distance from annotated exons are classified individually (LVP, CTM, RexD) and cumulatively (in all three strains) on the basis of RNA-seq read coverage in FPKM. **C)** functional classification of the 2025 NTUs found with similar coverage across the three strains. Abbreviations: cuticular protein (CP), immunity (IMM), metabolism (MET), oxido-reductase (REDOX), ribosomal protein (RP), transport (TR), related to transposons (TRANSPOSON), signal transduction (ST), transcription and translation (TT), unknown (UNK), unknown and blasting to hypothetical or conserved proteins from Anophelinae mosquitoes (UNK-A), *Culex quinquefasciatus* (UNK-CQ), Drosophilidae flies (UNK-D) and diverse functions (DIV).

RNA-seq reads supported the discovery of 10,321 novel transcriptional units (NTUs) following Cufflinks analysis using the *Ae. aegypti* genome annotation (AaegL1.2) as a guide. The majority of these NTUs (7475; 72.5%) mapped outside a window of 1000 base-pairs (bp) from the 5’- and 3’-end boundaries of annotated exons (Figure 3A). Many of the identified NTUs likely are unassigned extensions of exons or novel exons of genes annotated previously. However, 2482 NTUs mapped >10,000 bp away from annotated exons. Most *Ae. aegypti* introns are shorter than 10,000 bp (average intron length 4,685 nucleotides [nt]) [16] providing strong support for the conclusion that we identified unassigned and un-annotated transcriptional units, most likely novel genes. Further analyses of these 2482 NTUs show they are distributed over 1137 supercontigs, 531 of which had no previously annotated gene (Additional file 8: Table S6). The majority (64%) of the NTUs encode products of unknown functions (Figure 3C). Blast2GO searches attributed a putative function to ≤ 40% of the NTUs.

The predominant functional attributes were different for NTUs depending on their expression levels assessed by read coverage (Figure 3C). Transposon-related functions (transposon polyprotein, reverse transcriptase, and retrovirus-related *gag* and *env* polyproteins of the *gypsy* and *hms-beagle* retrotransposons) were the most-represented functional category among the 1702 NTUs with low read coverage (FPKM < 1) in all three strains (Figure 3B). Metabolism (carboxylpeptidase, carboxylate reductase, diacylglycerol kinase, pyrroline 5-carboxylate reductase, gamma-glutamyltranspeptidase and hydroxypyruvate isomerase) and signal transduction (receptors, zinc-finger proteins) were the most-represented categories among the 259 NTUs with FPKM < 10. Diverse functions and signal transduction were represented highest among the 53 NTUs with FPKM < 100. Only two proposed functions, lipoprotein receptor-related protein and a cytosolic large ribosomal subunit, could be assigned to the 11 NTUs with FPKM > 100.

## Discussion

We identified 131,764 non-redundant polymorphic sites in the *Ae. aegypti* transcriptome with at least one alternative nucleotide than what is present in the current genome annotation. Our approach included three different strains of *Ae. aegypti* and a large number of mosquitoes/strain (90 mosquitoes per RNA-seq library), allowing sampling of 180 distinct haploid genomes for each strain. The covered genetic diversity combined with a stringent definition of a SNP-gene (RNA-seq coverage over > 90% of their length, FPKM > 15 and present in all three strains) provide confidence that the polymorphisms identified represent legitimate allelic variations within and among strains. Furthermore, the discovery of 64 NTUs with FPMK ≥ 10 in supercontigs previously annotated as containing no protein-encoding genes increases the representation of our data set.

This transcript-based approach to SNP discovery is not a comprehensive survey of all genes because many may not have products represented in the specific tissues, stages and sexes of the selected samples. However, while our data show the under-representation of certain families of genes based on their expression profiles, the distribution of genes and SNPs across the annotated supercontigs supports the conclusion that we have a representative sample of the whole transcriptome. Differential gene ontology representation among the NTUs with respect to FPKM values most likely reflects the differences in samples sizes of clusters. The low representation of transposon-related transcripts supports the interpretation that these elements are not mobile in the developmental stages sampled.

The density of polymorphic sites in supercontigs and individual genes provides information that could be developed in analyses of selection pressures acting on each strain. Future approaches using single mosquitoes and correlating the variation identified in polyadenylated RNAs to the corresponding DNA could map genomic regions that are under positive or negative selection in context of whole-genome evolution. Our experimental design of applying RNA-seq to pooled RNA from a large number (90) of mosquitoes sampled at two different conditions (after blood-feeding and under sugar-feeding) was a cost-efficient way to obtain enough depth of coverage to call single nucleotide variations with confidence within a strain. However, having pooled samples prevent us to infer the genotype of each mosquito and consequently apply conventional analyses of molecular evolution to our data.

The average PS/kb identified in CTM and RexD is slightly lower than the density of SNPs per kb previously detected in a survey of polymorphisms across coding and non-coding regions of 25 genes in the Red-eye and Moyo-R strains of *Ae. aegypti*[22] and it is 1.8 fold higher than that detected in LVP. The AaegL1.2 genome annotation used for RNA-seq read mapping and SNP identification was derived from the LVP strain [16], as a consequence our LVP polymorphism data reflect only variability within the strain. The same value calculated from other strains reflects both the within-strain variability and the variation between each strain and LVP.

There was no general correlation between expression level and SNP density, as observed in yeast, fruit flies and salmon [23-25]. Polymorphisms resulting in synonymous substitutions were the most numerous. The second most-abundant type of polymorphism detected was in 3’UTRs. Nucleotide diversity was heterogeneous both in terms of number and position of SNPs, supporting the view of the transcriptome as a mosaic of components with different evolutionary histories [26].

The intragenic locations of polymorphisms in the SNP-genes associated with immunity varied with functional class. Polymorphisms were detected more frequently in the protein-encoding sequences of the CLIPs, serpin and heme-containing peroxidases (HPX). In contrast, SNPs were more prevalent in the UTRs of genes linked to autophagy, and ML-encoding genes. These findings are in agreement with a model in which some classes of immunity-related genes (for example, CLIPs, serpin and HPX) maintain common transcribed regulatory features while diversifying their specificity while other classes appear to conserve their specificity while accommodating different regulatory domains. Four of the five currently annotated thioredoxine peroxidase-encoding genes showed exclusively synonymous changes. These data correlate with hypotheses on the evolution of immunity-genes based on phylogenetic analyses that included Culicidae species and *Drosophila melanogaster*[27,28]. Thioredoxine peroxidases are thought to constitute the primary anti-oxidant system in insects, including *D. melanogaster*, *Anopheles gambiae* and *Ae. aegypti*, and their corresponding genes are conserved highly across mammals, fungi, worms and insects [29]. In contrast, HPX-encoding genes were found to be radiating faster with species-specific expansion through duplication in Culicidae species and *D. melanogaster*[27]. Limited gene duplication was detected among the autophagy genes in *D. melanogaster* and three mosquito species (*Ae. aegypti*, *An. gambiae* and *Culex quinquefasciatus*), supporting the interpretation that selective constraints may exist. CLIP and serpin-encoding genes are in large gene families showing recent diversification, with particular expansion in *Ae. aegypti*[27].

Polymorphisms were found predominantly in 3’- and 5’-UTRs in 5 of the 17 annotated *Ae. aegypti* MD2-like encoding genes. These data do not support phylogenetic analyses of MD2-like encoding genes in *Ae. aegypti* and *An. gambiae* that showed species-specific expansion and led to the hypothesis of a receptor activity for a broad spectrum of antigens for AgMDL1 of *An. gambiae*[27]. Functions and molecular mechanisms of MD2-like proteins have not been elucidated fully in mosquitoes and several MD-like proteins have altered gene expression following dengue infection in salivary glands or whole mosquito bodies [30,31].

SNP-genes included 19 of the 31 annotated sRNA members, 11 of which showed strain-specific SNPs. Representative members of the miRNA and siRNA pathways (LOQS, DCR2, FMR1, VIG and TSN) showed changes in UTRs more frequently than changes associated with non-synonymous substitutions. All but two of the non-synonymous changes identified in DCR2 across the LVP, CTM and RexD strains were identical to non-synonymous polymorphisms detected in wild-caught *Ae. aegypti* from Senegal, Mexico and Thailand and proposed to be under positive selection [17]. Three additional SNPs in exon 8 of DCR2 associated with synonymous mutations also were detected in *Ae. aegypti* from Thailand [32]. Laboratory-adapted strains of mosquitoes are expected to be less polymorphic than recent wild-derived lines due to genetic drift effects that result in reduced heterozygosity as a consequence of colony population structure. The similarity between the position of non-synonymous SNPs discovered in laboratory-adapted strains and wild-caught mosquitoes is an indirect validation of our data. The suite of SNPs presented here will be included in a larger SNP array that will increase the power of association-mapping analyses (Powell, personal communication) and greatly extend the number of molecular markers available for this species [5-7].

## Conclusions

We analyzed the transcriptomes of three *Ae. aegypti* strains, two that exhibit differential susceptibility to dengue-2 infection (CTM and RexD), and the strain (LVP) on which the available genome annotation is based. RNA-seq data generated from 90 mosquitoes of each strain supported the identification of 2482 NTUs, mostly with unknown functions, underscoring the need for continuous refinement of the current genome annotation.

We adopted a conservative approach to select genes for SNP analyses based on their RNA-seq coverage, requiring 90% minimum read coverage of each gene length, support for moderate expression level (FPKM > 15) and representation in all three strains. The identified 4492 “SNP-genes” that meet these criteria show an unbiased distribution across the genome and absence of functional clustering.

We further assessed the effect of the identified SNPs with respect to the annotated coding sequence and provide a summary of data for all SNP-genes. A dedicated analysis of immunity-related genes in the SNP-genes revealed differences in prevalence of types of polymorphisms according to functional categories, which mostly recapitulated results from phylogenetic analyses that included *Ae. aegypti*, *An. gambiae* and *D. melanogaster*, but also revealed differences between *Ae. aegypti* and *An. gambiae*. Validation of the methodology used here for SNPs characterization is provided by the concordance between non-synonymous substitutions detected in DCR2 in this and previous surveys of five wild *Ae. aegypti* populations.

The surge of dengue cases in the past 50 years, partly dependent on the expansion of its vectors species range, calls for urgent and innovative control measures [33]. Genetic-based control strategies represent a novel, rapidly-progressing approach [34]. Two transgenic lines have reached field-testing validation stage and two lines show a transmission-blocking phenotype in the laboratory [35-37]. The observed sequence polymorphisms and variation in expression profiles across different *Ae. aegypti* strains support the conclusion that a synthetic-approach for the development of effector genes would probably be more effective in achieving species-specificity, while maintaining efficacy across geographically-distinct populations [19,31,38,39].

## Methods

### Mosquito strains and rearing

Three *Ae. aegypti* laboratory strains, LVP, CTM and RexD, were used in this study. The origins of the three strains have been described previously [16,17,31,40,41]. Mosquitoes were reared in an insectary at 70-80% relative humidity, 28°C and with a 12-12 h light–dark photoperiod. Larvae were fed on a finely-ground fish food (Tetramin, Tetra Werke, Germany). Male and female mosquitoes were kept together in cages with unlimited access to water and sugar (raisins) until blood feeding. Mosquitoes aged 3–5 days after eclosion were allowed to feed on mice anaesthetized with a mixture of ketamine and xylazine. Forty-five females of each strain were maintained on a sugar-diet and frozen promptly at −80°C 5 hours after a bloodmeal. This time point was chosen based on previous studies showing extensive changes in gene expression across strains [19]. Vertebrate animals (for blood meals) were handled in strict accordance with the recommendations in the Guide for the Care and Use of Laboratory Animals of the National Institutes of Health and research protocols were approved by the Institutional Animal Care and Use Committee at the University of California, Irvine.

### RNA extraction and Illumina library preparation

RNA was extracted from pools of 3 female mosquitoes using the standard Trizol (Invitrogen) protocol. After verifying the quality of total RNA samples with an Agilent 2100 Bioanalyzer, 15 pools of sugar-fed and 15 pools of blood-fed mosquitoes, representing a total of 90 mosquitoes per strain, were combined in equal quantities for the preparation of a paired-end Illumina library. One library per strain was constructed by the DNA Technologies Core Facility at the UC Davis Genome Center [42]. Briefly, polyadenylated RNA was isolated from total RNA samples using oligo-d(T)25 magnetic beads (Dynabeads: Invitrogen, CA, USA). Once the dynabead-polyadenylated RNA binding was reversed chemically, the polyadenylated RNA was used as a template for first-strand synthesis that was converted subsequently to double-stranded cDNA. The resulting double-stranded overhang fragments are end-repaired by incubation in the presence of T4 DNA polymerase and Klenow polymerase. The polished fragments are phosphorylated by T4 polynucleotide kinase, followed by the addition of a single ‘A’ base to the 3’-end of the blunt-ended phosphorylated fragments. This ‘A’ base prepares the cDNA fragments for ligation to proprietary adapter oligonucleotides (Illumina single-read or paired-read sequences), which have a ‘T’ base at their 3’-end (Bio Scientific, USA). Library preparation followed the workflow protocol using the liquid handling Apollo 324 robot and PrepX DNA library preparation kit manufactured by InteGenX (Pleasanton, CA). Ligation products were purified and subjected to a final gene amplification step (10 cycles).

Amplified libraries were assessed quantitatively and qualitatively by Nanodrop ND-1000 (Thermo Scientific, DE, USA) UV/Vis spectroscopy, DNA bioanalyzer 2100 (Agilent, CA, USA) microfluidics, and real-time quantitative gene amplification (Kappa Biosystem, USA) to determine sequence-able molecules for pooling of libraries at equimolar concentration and the subsequent sequencing on Illumina HiSeq for paired reads of 100 bases.

### RNA-seq data analyses

Raw Illumina RNA-seq reads from each sample were processed initially to remove 3’-end adapter contamination and low-quality sequences (phred-scaled quality score < 20) with custom software (Scythe and Sickle, available at https://github.com/ucdavis-bioinformatics). The trimmed reads were aligned first to the *Ae. aegypti* reference transcriptome fasta (AaegL1.2 from VectorBase), using the short-read aligner BWA, to calculate insert size for each sample [43]. TopHat v.2.0.4 [44] was used to align spliced representations of all reads of each strain to the *Ae. aegypti* supercontigs, with the AaegyL1.2 basefeatures gtf as a guide. TopHat output, comprising exclusive and unambiguously-mapped reads, was the starting point for all subsequent analyses. The cuffmerge and cuffcompare modules within Cufflinks v.2.0.2 [45] were run, using the AaegyL1.2 basefeatures gtf as an annotation guide and allowing the discovery of NTUs, to generate new gtf and transcript fasta files. The NTUs were annotated using Blast2GO [46,47]. Cufflinks also was used to calculate the accumulation levels of poly-adenylated RNAs as FPKM. The TopHat alignments were analyzed by coverageBed (BEDTools v.2.16.2 [48] to count the number of reads per genome base location. The coverageBed output was filtered with a custom script to calculate coverage by gene for all bases with a minimum coverage threshold requirement of three reads. Freebayes v. 0.9.4 [49] was run to call variants simultaneously for all three strains. The freebayes vcf output was post-processed with custom scripts to include only those locations for each genotype containing variants with coverage and SNP quality meeting specific criteria: minimum SNP QUAL 40, genotype minimum QA (sum of quality of the alternate observations) 40, minimum DP (read depth) 3, and minimum AO (alternate allele observations) 2. SnpEff 3.0 [50] was run to predict the effects of the variants in the processed Freebayes vcf files. Gene function was predicted through the Biomart function in EnsamblMetazoa [51].

### Availability of supporting data

RNA-seq data are deposited at NCBI’s Sequence Read Archive (SRA) under accession numbers SRX253218 (CTM), SRX253219 (RexD), SRX253220 (LVP). The mapping coordinates, Blast description, coverage and SNPs identified for the 2482 Novel Transcriptional Units are provided in Additional file 8: Table S6. The data also are available at the CalTech *Aedes aegypti* expression browser http://www.Aedes.caltech.edu.

## Competing interests

No financial competing interests.

## Authors’ contributions

MB, MBt, JF, OM and WAD performed all analyses; MB, OM, AAJ conceived the study; MB, OM, MBt and AAJ wrote the manuscript. All authors read and approved the final manuscript.

## Supplementary Material

Additional file 1: Figure S1 SNP genes in each strain. Click here for file

Additional file 2: Table S1 SNP-genes. Click here for file

Additional file 3: Table S2 Comparisons of the numbers of annotated and SNP-genes in each supercontig. Click here for file

Additional file 4: Figure S2 Number of polymorphic sites/kb and depth of read coverage. Click here for file

Additional file 5: Table S3 No SNPs genes. Genes meeting SNP-gene criteria but with no SNPs. Click here for file

Additional file 6: Table S4 Position and effect of SNPs on ORFs and in UTRs. Click here for file

Additional file 7: Table S5 List of immunity genes and positions and effects on ORFs and in UTRs. Click here for file

Additional file 8: Table S6 List and properties of the 2482 Novel Transcriptional Units. Click here for file
