# Supplementary material for: Probing functional polymorphisms in the dengue vector, Aedes aegypti
Source: BMC Genomics. 2013 Oct 29;14:739. doi: 10.1186/1471-2164-14-739 (PMC4007706; doi:10.1186/1471-2164-14-739)
Supplement: Additional file 1: Figure S1 — SNP genes in each strain. [file 1471-2164-14-739-S1.pdf]

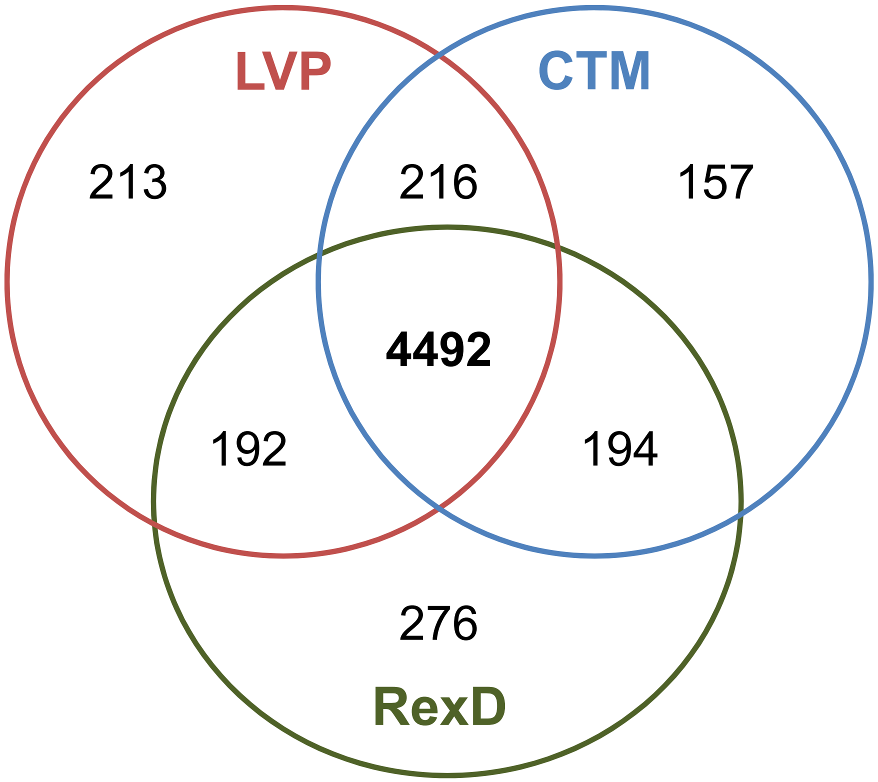

Figure S1. Numbers of SNP genes in each strain. Venn diagram showing the number of genes that had read coverage over >90% of their length and depth of coverage of ≥15 FPKM in all three strains. Abbreviations CTM, Chetumal; LVP, Liverpool; RexD, Rexville-D.
