# Supplementary material for: Probing functional polymorphisms in the dengue vector, Aedes aegypti
Source: BMC Genomics. 2013 Oct 29;14:739. doi: 10.1186/1471-2164-14-739 (PMC4007706; doi:10.1186/1471-2164-14-739)
Supplement: Additional file 4: Figure S2 — Number of polymorphic sites/kb and depth of read coverage. [file 1471-2164-14-739-S4.pdf]

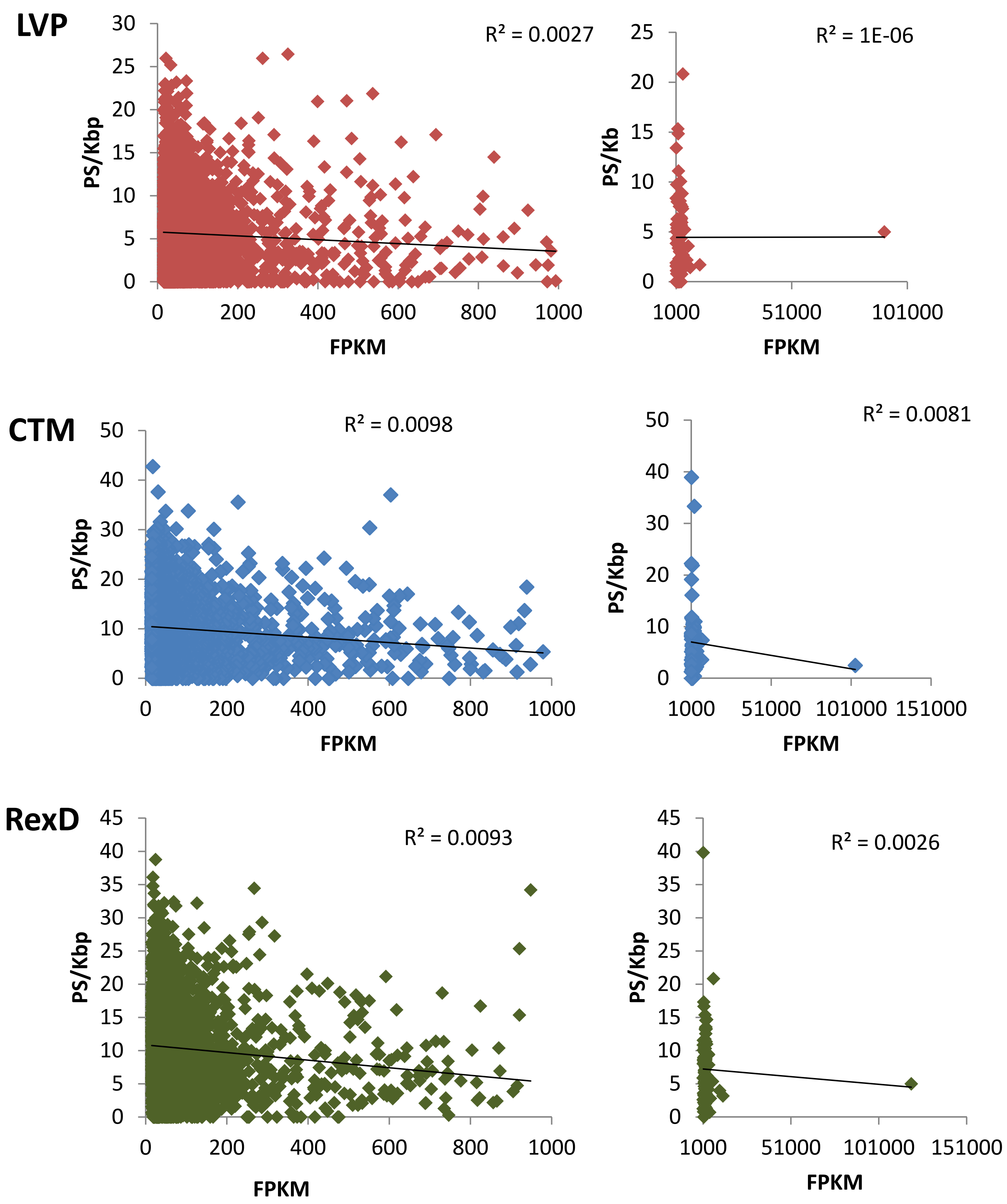

Figure S2. Number of polymorphic sites/kb and depth of read coverage. Regression analyses between the density of polymorphic sites and the FPKM value of each SNP-gene. Abbreviations CTM, Chetumal; LVP, Liverpool; RexD, Rexville-D.
